# Comparison of genotoxic impurities in extracted nicotine vs. synthetic nicotine

**DOI:** 10.3389/fchem.2024.1483868

**Published:** 2024-10-14

**Authors:** Ayesha Nisathar, Hui Chen, Xiaoli Lei, Zeyu Zeng, Jia Chen

**Affiliations:** ^1^ Analytical Research Development and Quality Control, JSTAR Research Inc., Cranbury, NJ, United States; ^2^ Porton Pharma Solutions, Chongqing, China

**Keywords:** chromatography, comparison, extraction, nicotine, nitrosamine, purity and impurity, synthetic

## Abstract

Nicotine is a chiral alkaloid; nitrogen-containing organic compound that occurs naturally. (S)-nicotine is extracted from Tobacco plants and used as the key addictive ingredient in many smoking products. Synthetic nicotine has gained the interest of many smoking product manufacturers over the last few decades due to the ease and low cost of manufacturing. Another claimed advantage of synthetic nicotine is the absence of genotoxic impurities that form during the extraction process of nicotine. These impurities are other plant alkaloids, phenolic compounds, and heavy metals. Additionally, the U. S. FDA has implemented new regulations on the quality control of synthetic nicotine. However, only a very few research articles have been published on assessing the complete impurity profile of synthetic nicotine. Therefore, the need to know the composition difference between tobacco-extracted nicotine vs. synthetic nicotine is highly necessary. In this research study, the impurity profile of thirteen different lots of synthetic nicotine was compared with fourteen lots of nicotine extracted from plants using in-house analytical methods. First, the samples were tested for other alkaloids and phenols by reversed-phase High-Performance Liquid Chromatography (HPLC). Second, the chiral purity was analyzed by normal phase HPLC. Third, lead and arsenic content were tested by atomic absorption and fluorescence spectrometry. Fourth, nicotine-specific nitrosamines were tested by LC-MS. The reversed phase HPLC data suggested similar quantities of total impurities in both synthetic and tobacco-extracted nicotine (0.1%). However, synthetic nicotine lacks some impurities such as cotinine, nornicotine, and nicotine-N-oxide. Additionally, the synthetic nicotine lots used in this study have high enantiomeric purity similar to the tobacco-extracted nicotine.

## Introduction

**SCHEME 1:**
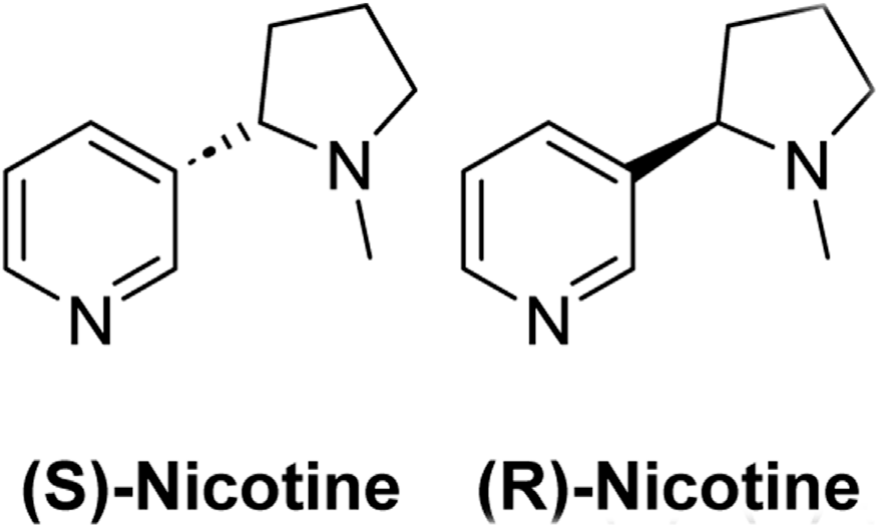
Structures of (S) and (R)-Nicotine.

Nicotine is an alkaloid; nitrogen-containing organic compound that occurs naturally in many plant varieties and is the key component in tobacco plants. Structurally, nicotine is a chiral molecule with a single stereocenter (Scheme 1) (Henningfield et al., 2009). Nicotine is extracted from tobacco plants and used as the key ingredient in many smoking products, such as cigarettes, cigars, smokeless tobacco, hookah tobacco, and most e-cigarettes (Robinson and Pritchard, 1992).

The (S)-nicotine is known to be present in excess in tobacco plants and is considered a highly addictive form. While (R)-nicotine is found in less amounts in tobacco plants and considered less addictive, proper evidence confirming its addictiveness is less.

Another method to generate nicotine is chemical synthesis. Synthetic nicotine has gained the interest of many Tobacco product manufacturers over the last few decades due to the minimal regulations imposed by the FDA on synthetic Nicotine compared to tobacco plant-derived Nicotine. However, the FDA has recently implemented new controls on synthetic Nicotine. An additional advantage of synthetic Nicotine is that it the less impact on the environment such as an increased global footprint. Zafeiridou et al. evaluated the environmental impacts of Tobacco cultivation and estimated 0.2% of CO_2_ global emissions from Tobacco cultivation (Zafeiridou et al., 2018). Furthermore, the Tobacco plant affects the lifespan of crop-friendly bacteria/fungi and hence causes a subsequent effect on maize crop growth (Lisuma et al., 2019). The composition of tobacco-extracted nicotine varies depending on the farming practices, climate, and which part of the plant leaf is harvested. Lisko et al. reported that many tobacco-derived nicotine products have discrepancies between labeled and actual nicotine concentrations. Furthermore, many common products contain impurity alkaloids at concentrations that exceed USP limits for impurities (Lisko et al., 2015).

The leading manufacturers of Synthetic Nicotine are Zanoprima Lifesciences, NJOY, Contraf-Nicotex-Tobacco, and Next Generation Labs. The enantiomeric purity of the final Nicotine of most of these manufacturers is poor (≤50%) and often requires a stereoselective purification step which reduces the yield of (S)-Nicotine (Jordt, 2023). Synthetic Nicotine has been used in many e-cigarettes, Nicotine pouches, and many other Tobacco products. Tobacco product manufacturers have marketed these as safe alternatives to Tobacco extracted Nicotine and attracted many younger populations (Ma et al., 2023). However, whether Synthetic Nicotine is a healthier alternative compared to Tobacco extracted Nicotine is still under debate (Kowitt et al., 2023). Porton Pharma Solutions in China has recently patented a novel Nicotine synthetic pathway that produces more of the (S)-Nicotine (Lin, 2023). The goal of this work is to compare the impurities in the Nicotine in the novel synthetic pathway with several lots of Tobacco extracted Nicotine.

According to a fact sheet released by the U. S. Department of Food and Drug Administration (FDA), other chemicals in Tobacco or Tobacco products cause more health effects than Nicotine itself. The statement is that “It’s the thousands of chemicals contained in tobacco and tobacco smoke that make tobacco use so deadly. This toxic mix of chemicals—not nicotine—causes serious health effects, including fatal lung diseases and cancer” (Talhout et al., 2011). Better quality control in synthetic nicotine products is required on a global scale to ensure that customers can safely use them. Common impurities reported in tobacco-extracted nicotine are naturally occurring other alkaloids, nicotine enantiomers, nicotine-specific nitrosamines, and heavy metals.

**SCHEME 2:**
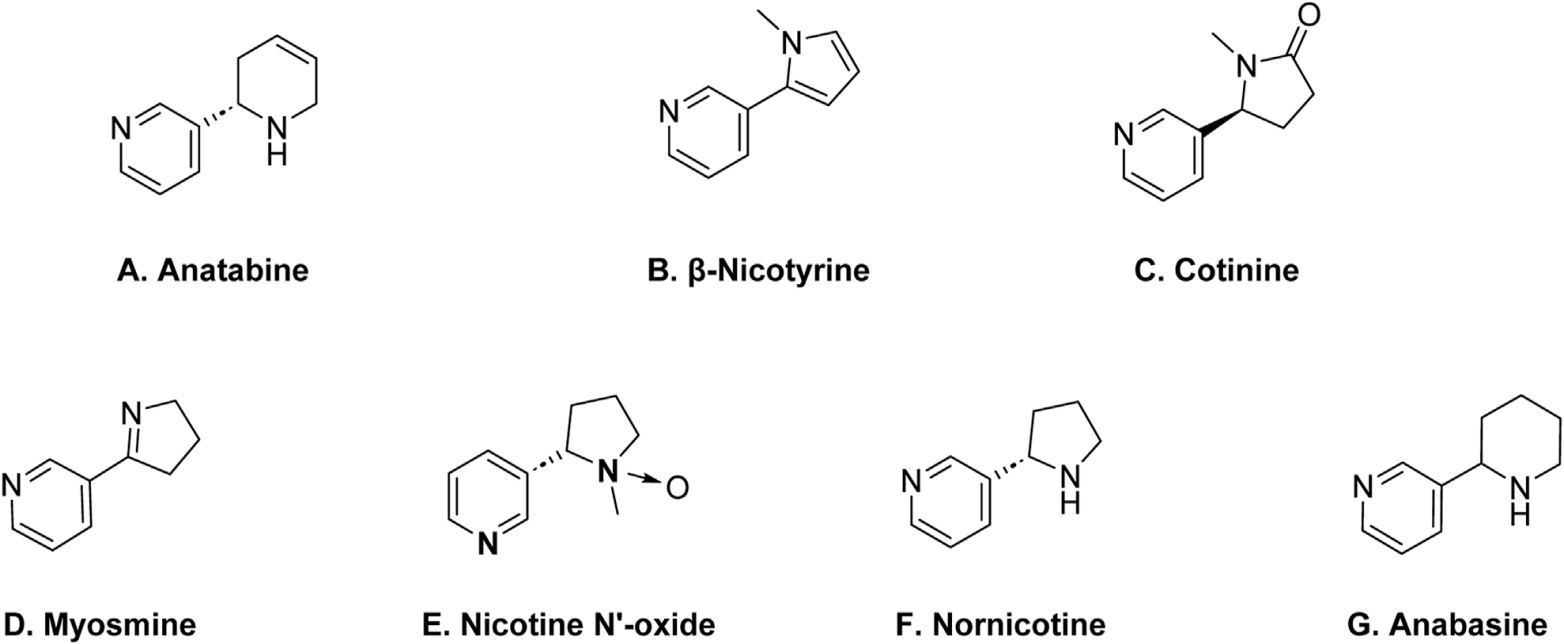
Structures of key alkaloid impurities in nicotine; **(A)** Anatabine, **(B)** β-Nicotyrine, **(C)** Cotinine, **(D)** Myosmine, **(E)** Nicotine N’-oxide, **(F)** Nornicotine, **(G)** Anabasine.

Anatabine, β-nicotine, cotinine, myosmine, nicotine-N′-oxide, nornicotine, and anabasine are the main naturally occurring alkaloid impurities found in nicotine (Scheme 2) (Henningfield et al., 2009). Anatabine is structurally distinct from nicotine. β-nicotine is structurally related to nicotine and is considered a derivative of nicotine. The prefix “β-” indicates the position of a specific chemical bond in the molecule. Chemically, β-nicotyrine is an isomer of anatabine. Cotinine is another alkaloid impurity in nicotine, and it is also a metabolite of nicotine. Cotinine is formed when the body metabolizes nicotine, and it can also be present in tobacco. Myosmine is also structurally related to nicotine. Nornicotine is an isomer of nicotine, meaning it has the same molecular formula but a different arrangement of atoms. Anabasine is a pyridine alkaloid which is structurally related to nicotine. All of these alkaloid impurities are often present in relatively small amounts compared to nicotine, but their presence contributes to the overall alkaloid profile of tobacco (Avagyan et al., 2021).

**SCHEME 3:**
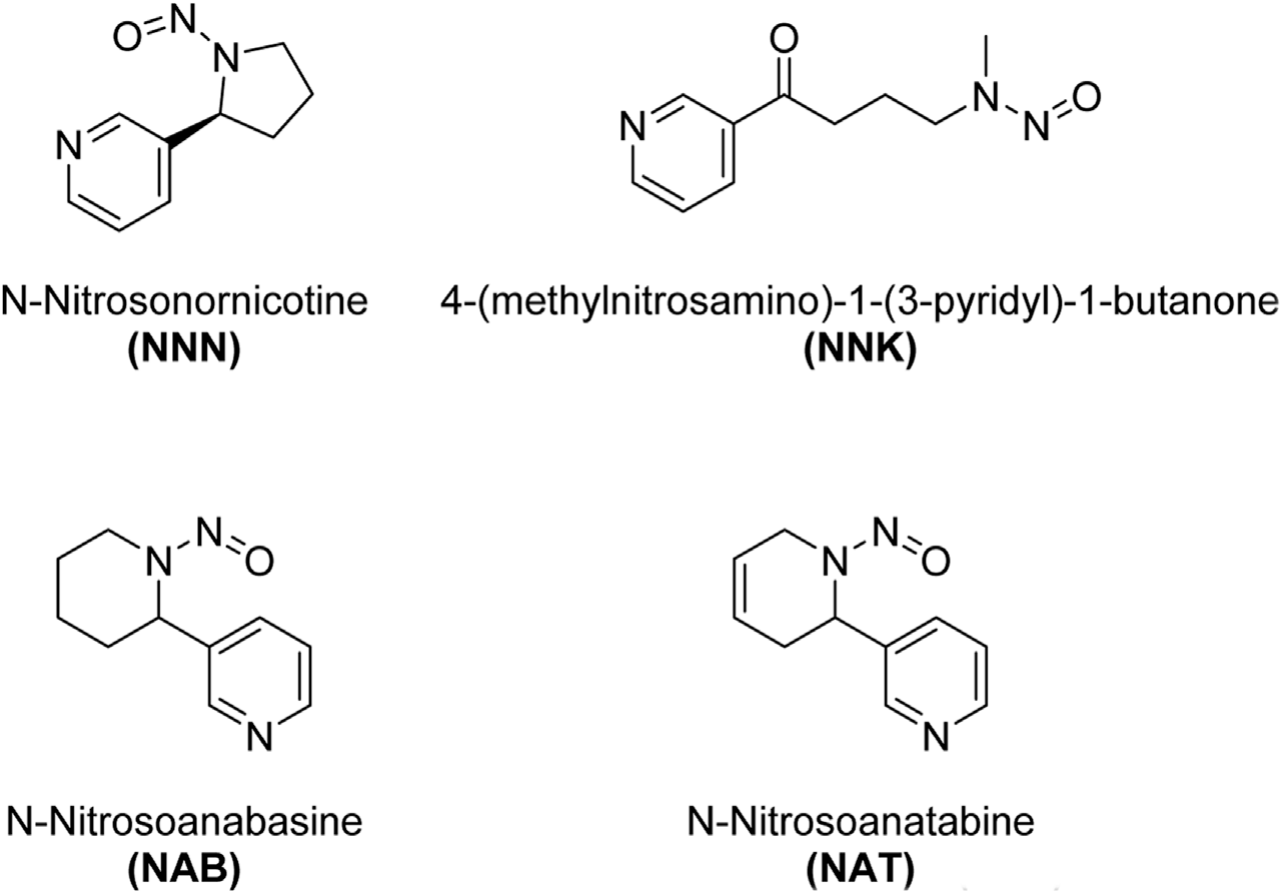
Structures of the most common nitrosamines found in nicotine.

Tobacco-specific N-nitrosamines (TSNAs) are a group of chemical compounds that are found in tobacco and tobacco smoke. N-nitrosamines are known carcinogens, meaning they have the potential to cause cancer. TSNAs are formed through a specific chemical process during the growth, curing, and processing of tobacco, as well as during the combustion of tobacco, which occurs when smoking (Konstantinou et al., 2018). Some of the key tobacco-specific N-nitrosamines are shown in Scheme 3. These include N′-Nitrosonornicotine (NNN), 4-(Methylnitrosamino)-1-(3-pyridyl)-1-butanone (NNK), N-Nitrosoanabasine (NAB), N-Nitrosoanatabine (NAT).

N′-Nitrosonornicotine (NNN): NNN is a nitrosamine compound that is formed during the curing and processing of tobacco, as well as during the smoking of tobacco products. It is known to be carcinogenic. 4-(Methylnitrosamino)-1-(3-pyridyl)-1-butanone (NNK): NNK is another important nitrosamine found in tobacco and tobacco smoke. It is known to be a potent lung carcinogen. N-Nitrosoanabasine (NAB): NAB is another nitrosamine compound related to nicotine that is found in tobacco. N-Nitrosoanatabine (NAT): NAT is a nitrosamine found in tobacco and tobacco smoke. It is a derivative of nicotine, and its presence has been identified in both unburned tobacco and mainstream smoke.

Due to the association of TSNAs with cancer, efforts have been made to reduce their levels in tobacco products and to develop methods to minimize their formation during the tobacco manufacturing process (Yalcin and de la Monte, 2016). Additionally, public health campaigns aim to raise awareness of the health risks associated with tobacco use, including exposure to TSNAs through smoking.

The goal of the current research is to identify impurities in synthetic nicotine and extracted nicotine and justify the claims made by synthetic nicotine manufacturers. Additionally, synthetic nicotine used for this study is from in-house synthesized nicotine from a recently patented synthetic procedure. A similar work has been published by Cheetham and co-workers from “Enthalpy Analytical LLC” in 2022. Enthalpy analytical screened only 5 lots of synthetic Nicotine and 5 lots of Extracted Nicotine. In this study, we screened 13 different lots of Synthetic Nicotine and compared the results with 14 different lots of extracted nicotine (Cheetham et al., 2022).

Analytical methods were used to quantify the impurities in the selected Nicotine lots. European Pharmacopeia (Eu. P. 10.0) monograph method was followed for assay, purity, and impurity analysis using potentiometry and High-Performance Liquid Chromatography (HPLC) instruments, respectively. An in-house developed normal phase chiral HPLC with Diode Array Detector was used to determine enantiomeric purity. Quantification of nitrosamines was done using liquid chromatography-mass spectrometry (LC-MS). Lead and Arsenic content quantification was done using Atomic Absorption Spectrophotometer (AAS) and Atomic Fluorescence Spectrophotometer, respectively.

## Materials and methods

Thirteen lots of synthetic Nicotine from Porton China in-house was taken and fourteen different Extracted Nicotine from local vendors in China was used. The names of the vendors will not be provided herein due to business cooperation confidentiality. Lot numbers of Synthetic Nicotine and Extracted Nicotine are provided in Tables 1, 2 below. Each lot number is abbreviated for simplicity and the respective abbreviations along with the actual lot number for synthetic nicotine and extracted nicotine batches are listed in Tables 1, 2, respectively.

**TABLE 1:** Synthetic nicotine lot numbers and respective abbreviations.

Synthetic nicotine actual lot #	Abbreviation
205351-305-00011-P2-01	SyN-1
205351-305-00012-P2-01	SyN-2
205351-305-00013-P2-01	SyN-3
HC-1	SyN-4
HC-2	SyN-5
HC-3	SyN-6
HC-4	SyN-7
HC-5	SyN-8
HC-6	SyN-9
HC-7	SyN-10
HC-8	SyN-11
HC-9	SyN-12
HC-10	SyN-13

**TABLE 2:** Extracted nicotine lot numbers and respective abbreviations.

Extracted nicotine actual lot #	Abbreviation
103700-304-00006	ExN-1
103700-305-00051	ExN-2
HN-20230704	ExN-3
HN-2023062202	ExN-4
TQ-1	ExN-5
TQ-2	ExN-6
TQ-3	ExN-7
TQ-4	ExN-8
TQ-5	ExN-9
TQ-6	ExN-10
TQ-7	ExN-11
TQ-8	ExN-12
TQ-9	ExN-13
TQ-10	ExN-14

### Nicotine content/assay

Nicotine assay was performed according to European Pharmacopeia (Eu. P. 10.0) reference standard testing procedure. Nicotine-free base content was analyzed potentiometrically using a Mettler Toledo T5 titrator. 60 mg of the test product was accurately weighed into a 50 mL clean dry beaker and 40 mL of glacial acetic acid was added to fully dissolve. The sample was titrated with perchloric acid titration solution (0.1 mol/L) until the electrode potential stabilized as the endpoint. Each titration was conducted in duplicate. Glacial acetic acid solution without sample was titrated with perchloric acid titration solution (0.1 mol/L) until the electrode potential stabilizes as the endpoint and is used as the blank reading. Calculations were done based on the facts and the EU. P. statement that corresponds 1 mL of 0.1 mol/L perchloric acid titration solution to 0.00811 g of C_10_H_14_N_2_ nicotine-free base.

### Purity and related substances

Analysis of impurities present in Nicotine samples was conducted using HPLC according to Eu. P. 10.0 specifications. Agilent 1200 instrument with a DAD detector was used. Nicotine for system suitability chemical reference standard (CRS) batch 5 (catalog number Y000123, SDS product code 202300056) was used as the reference standard for the assay. The nicotine system suitability solution and nicotine test solutions were prepared according to EU guidelines.

### Isomeric purity

In-house synthesized Nicotine racemate oxalate (MT117) was used as a marker to test enantiomeric purity. Agilent 1260 instrument with UV detector was used and Diacel CHIRALCEL OD-H 4.6 × 250 mm, 5 µm (part number 14325) column was used. HPLC method condition was an isocratic gradient with n-hexane-ethanol = 95:5 (v/v) mobile phase, flow 1.0 mL/min, and total run time 20 min. UV detection wavelength at 260 nm was used. The sample injection volume was 10 μL. n-hexane-ethanol = 95:5 (v/v) was used as the diluent/blank. The Isomer marker solution was prepared by weighing 50 mg of MT117 racemate oxalate and dissolved in 1 mL of 20% NaOH aqueous solution. Then, extracted it with 1.5 mL of n-hexane. The extracted MT117 racemic mixture in n-hexane solution was diluted 10 times with diluent and injected as an isomer marker solution. Nicotine sample solutions were prepared by dissolving 20 mg of the test sample in a 20 mL volumetric flask with a diluent. All the solutions were shaken well and sonicated to mix well before use.

### Nicotine specific nitrosamine testing

LCMS-MRM (multiple reaction monitoring) was used for qualitative and quantitative analysis of nitrosamines. The method was adopted from the Nicotine regulatory for China. The tested N-nitrosamines are N'-Nitrosonornicotine (NNN), 4-(Methylnitrosamino)-1-(3-pyridyl)-1-butanone (NNK), N-Nitrosonornicotine (NAT), N-Nitrosoanabasine (NAB). Deuterated N-Nitroso Nicotine (NNN-d4), Deuterated 4-sulfur nitrosamine) -1-(3-pyridinyl) -1-butone (NNK-d4), Deuterated Nitrosonornitine (NAT-d4), and deuterated (R, S)-N-Nitrostobasine (NAB-d4) was used as internal standards for NNN, NNK, NAT, and NAB respectively. Method details and sample preparation are described in the Supplementary Section S1.

### Metal analysis

#### Lead (Pb)

Atomic Absorption Spectrophotometer, SP-3803AA, 2020-JS001 Shanghai Spectral Instrument Co., Ltd. was used for Pb analysis. Instrument parameters used for testing are described in the Supplementary Material. Pb standard solution of 1,000 mg/L from Guobiao (Beijing) Testing & Certification Co. Ltd. (lot#221008-7-1) was used to prepare a Pb standard curve of 0, 2, 5, 10, 15, 20 μg/L by diluting with Milli-Q water. The nicotine sample for testing was prepared by dissolving 0.1 g of accurately weighed sample in a beaker using nitric acid (7 mL), sulfuric acid (5 mL), perchloric acid (4 mL), and hydrogen peroxide (2 mL). 0.5 mL of this solution was added into a 25 mL volumetric flask and diluted up to the mark with Milli-Q water. The sample was shaken before testing.

#### Arsenic (As)

Arsenic content was analyzed by Atomic Fluorescence Spectrophotometer AFS-933 instrument. A standard solution of 100 mg/L from the Environmental Standard Institute (lot#103017-1) was used to prepare a standard dilution series of 0, 1, 2, 5, and 10 μg/L using Milli-Q water as a diluent and a linearity curve of As concentration vs. fluorescence was plotted. Nicotine samples for testing were prepared in the same manner as Pb sample preparation.

## Results

### Assay

**SCHEME 4:**
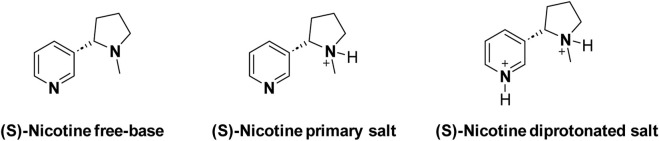
Forms of nicotine.

Assay was conducted to determine the “free base form” content in given Nicotine lots. The free base assay in extracted nicotine serves the purpose of quantifying the amount of nicotine in its free base form within a given sample. Nicotine exists in two primary forms: free base and salt. The free base form is the unprotonated, neutral state of nicotine, while the salt form results from the combination of nicotine with an acid (Duell et al., 2018). A third form of diprotonated nicotine is another possible structure (Scheme 4). However, there’s a lack of evidence to support the existence of the diprotonated nicotine form in any smoking products (El-Hellani et al., 2015; Pankow et al., 2003).

**FIGURE 1:**
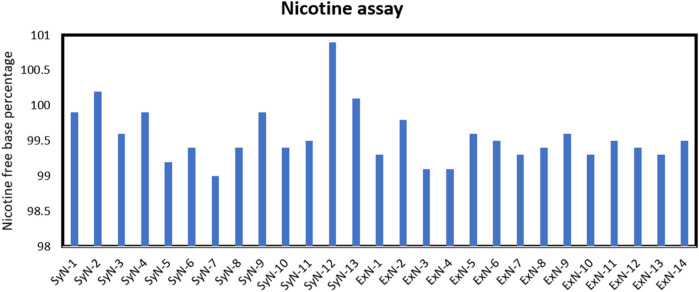
Nicotine assay percentage for synthetic and extracted nicotine lots.

In the context of both synthetic and extracted nicotine, especially in the tobacco industry or related fields, the free base assay is crucial for several reasons. Nicotine in its free base form is more readily absorbed by the body than its salt forms (Talih et al., 2020). Therefore, knowing the concentration of free-base nicotine provides valuable information about the potential physiological impact of a given nicotine-containing product. Nicotine manufacturers use the free base assay to ensure product quality/product performance in nicotine formulations such as e-cigarettes, nicotine patches, or gums. Lastly, regular monitoring of free-base nicotine content is not only a part of quality control processes in the tobacco industry but also ensures compliance with regulations and standards. It also helps maintain product consistency and ensures that consumers receive the expected nicotine levels when using a particular product. The assay methods for determining free base nicotine levels often involve chemical analysis, such as titration or chromatography techniques (Gholap et al., 2020). Eu. P. specified potentiometric method was used to determine the free base content in synthetic and extracted nicotine samples. The nicotine-free base percentage of all the lots is tabulated in Figure 1. Both synthetic and extracted nicotine had assay percentages greater than 99.1% making them identical in nicotine form content.

### Related impurities

**FIGURE 2:**
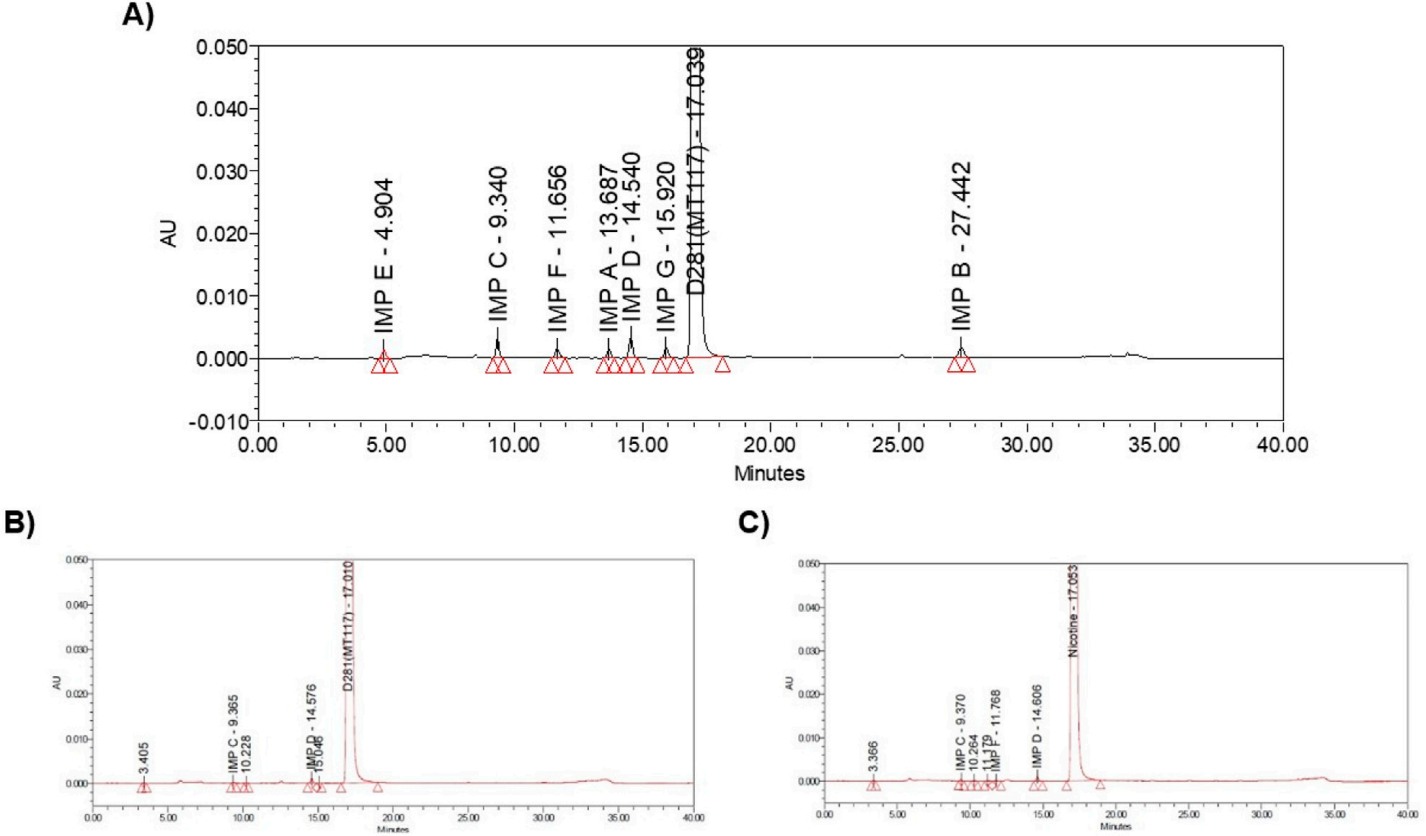
**(A)** Nicotine system suitability solution, **(B)** SyN-1 achiral chromatogram, **(C)** ExN-1 achiral chromatogram.

The European Directorate for the Quality of Medicines & Health (EDQM) classifies 7 impurities (A, B, C, D, E, F, G) in the Nicotine for system suitability chemical reference standard (CRS) and hence used as an impurity’s marker. The impurities are A: anatabine, B: β-nicotyrine, C: cotinine, D: myosmine, E: nicotine N′-oxide, F: nornicotine, G: anabasine. The Nicotine CRS chromatogram showing all labeled impurities is shown in Figure 2A. The retention times of impurities are A: anatabine, B: β-nicotyrine, C: cotinine, D: myosmine, E: nicotine N′-oxide, F: nornicotine, G: anabasine 13.687, 27.442, 9.340, 14.540, 4.904, 11.656 min, respectively. HPLC system suitability was assessed before sample analysis and ensured Eu. P. criteria were met. EU. P. system suitability criteria is to have a minimum resolution of 2.5 between impurity peak G and Nicotine. The system suitability injection sequence includes two blanks, and six reference standard solutions. In addition to the EU. P. system suitability criteria satisfaction, the relative standard deviation (RSD) was checked and ensured that it is within the standard Pharmacopeial range (RSD of ≤35.0% for peaks with area percentage 0.05%–0.10%, RSD of ≤20.0% for peaks with area percentage 0.10%–0.50%, RSD of ≤10.0% for peaks with an area percentage 0.50%–1.00%). A sample chromatogram from one synthetic nicotine lot and one extracted nicotine are provided in Figures 2B, C, respectively.

**FIGURE 3:**
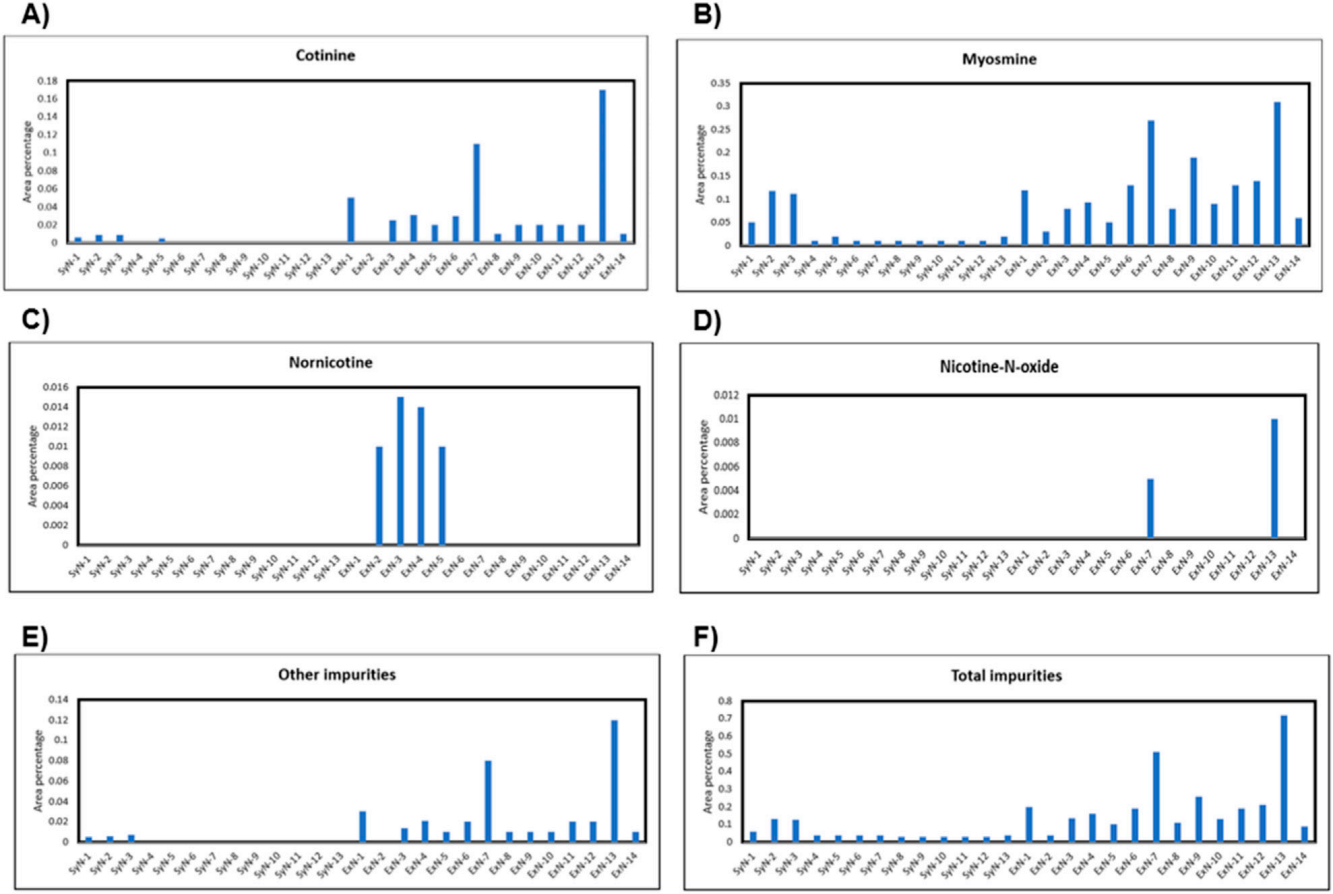
Impurity percentage in each nicotine lot vs. synthetic nicotine and extracted nicotine lot number for different impurities; **(A)** cotinine, **(B)** myosmine, **(C)** nornicotine, **(D)** nicotine-N’-oxide, **(E)** other impurities, **(F)** total impurities.

All the known impurities including other impurity peaks in the chromatograms were integrated according to Eu.P. guidelines and each impurity percentage content both synthetic and extracted nicotine was tabulated, and depicted in Figures 3A–F. The area percentage of impurity cotinine vs. different synthetic and extracted nicotine lots are shown in Figure 3A. Similarly, area percentage of myosmine, nornicotine, nicotine-N-oxide, other impurities, and total impurities were plotted against different Nicotine lots and shown in Figures 3B–F, respectively. Anatabine, β-nicotyrine, and anabasine were not detected in any of the synthetic or extracted nicotine batches. Naturally occurring other alkaloids (cotinine, nornicotine, nicotine-N-oxide) were not found or found less in synthetic Nicotine. However, overall, the reversed-phase HPLC data suggested similar quantities of total impurities in both Synthetic and tobacco extracted Nicotine (0.1%).

### Enantiomeric purity

**FIGURE 4:**
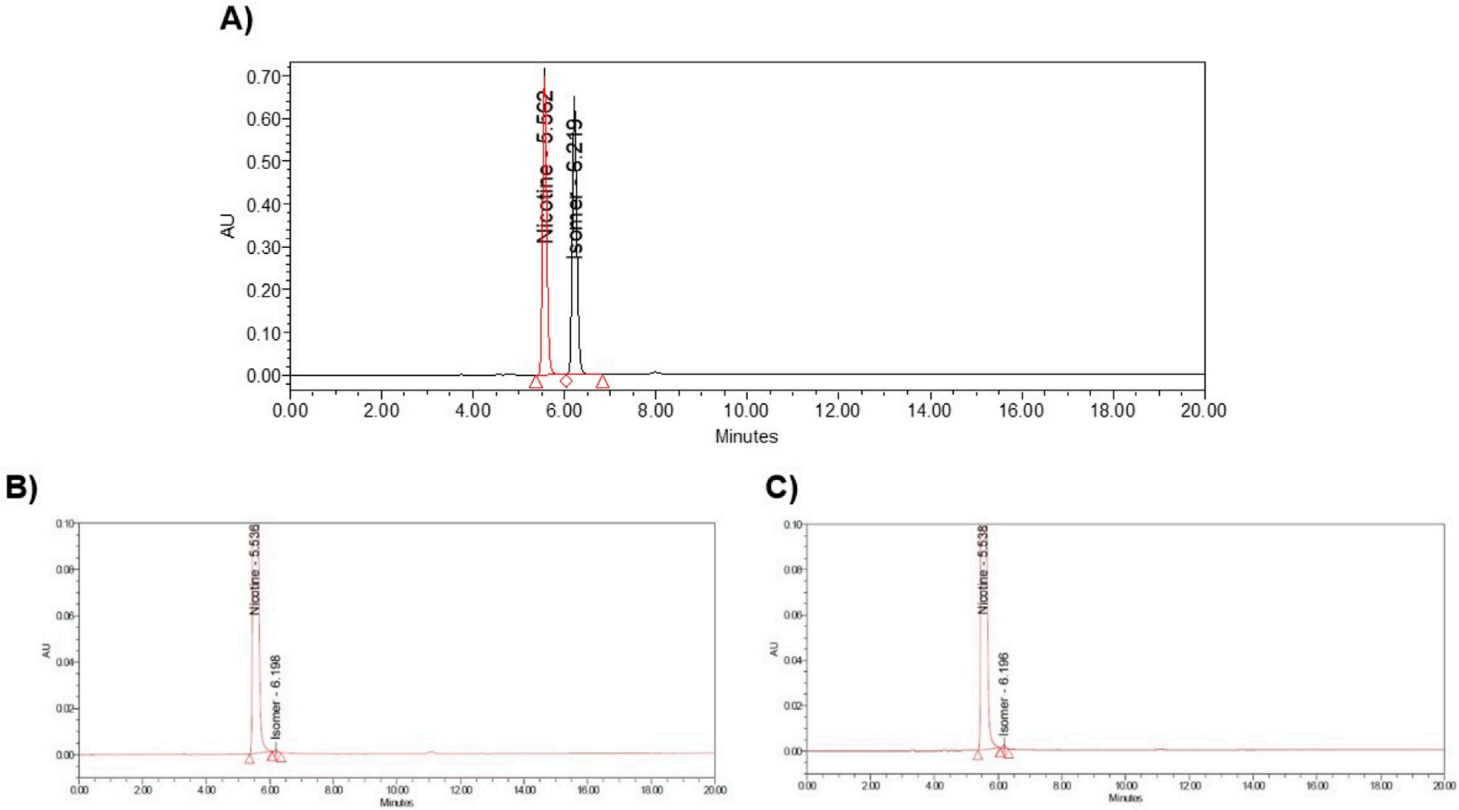
Example chromatograms of **(A)** MT117 nicotine (S) and (R) racemic marker solution, **(B)** Syn-1 chiral chromatogram, and **(C)** ExN-1 chiral chromatogram.

**FIGURE 5:**
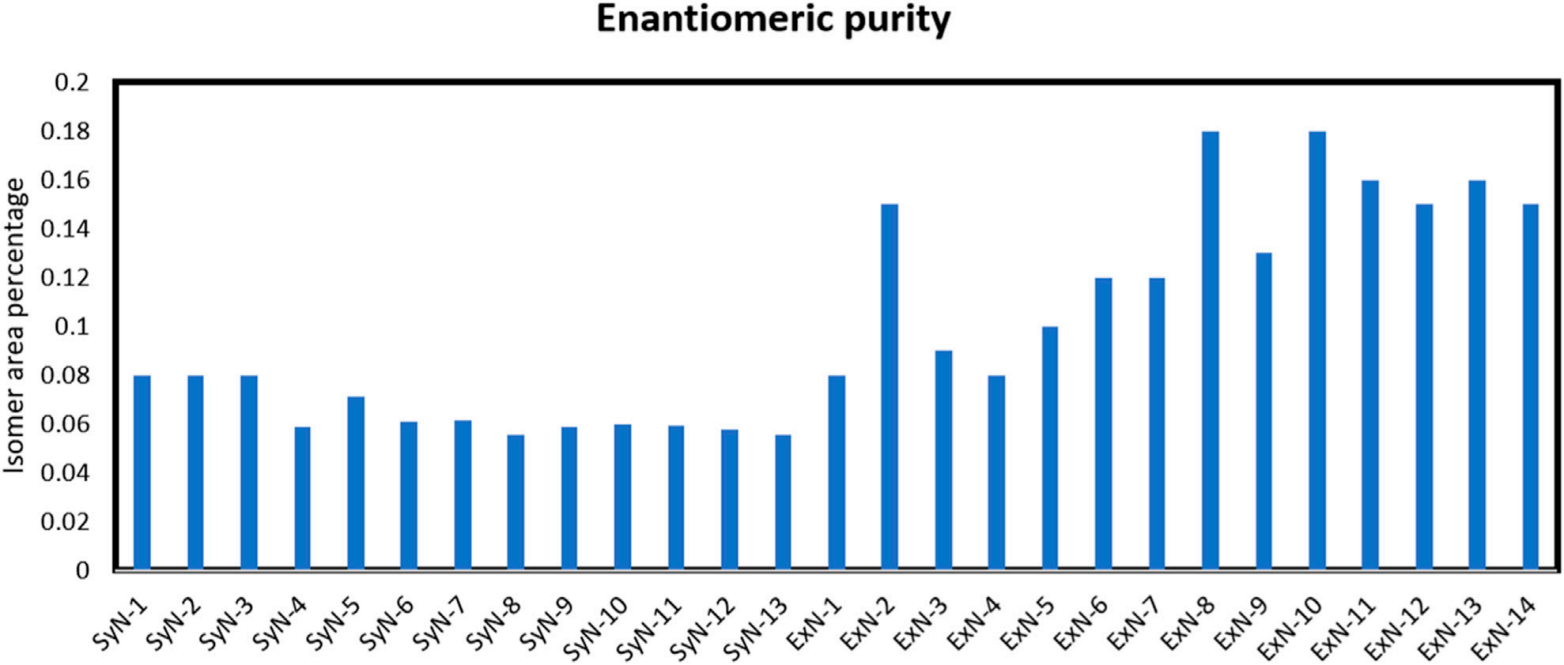
(R)-Nicotine enantiomer area percentage in synthetic and extracted nicotine lots.

(S)-Nicotine and (R)-Nicotine retention times are 5.562 and 6.219 min respectively (Figure 4A). The resolution between the two isomers was 3.9. Two sample chiral chromatograms for one synthetic nicotine and extracted nicotine are shown in Figures 4B, C, respectively. Synthetic Nicotine lots used in this study have high enantiomeric purity similar to the Tobacco extracted Nicotine (Figure 5).

### Nicotine nitrosamines

Nitrosamines were not detected in any synthetic or extracted nicotine lots. See Supplementary Section S1 for details on standard curve preparation, linearity curve, sample chromatograms, and nitrosamine content results for all the samples.

### Metal analysis

Lead and arsenic are heavy metals that can pose serious health risks when present in significant amounts. In the context of synthetic and extracted nicotine, it’s crucial to monitor and control the levels of these contaminants, as they can have harmful effects on human health. Extracted nicotine most likely accumulates heavy metals through soil/fertilizers and synthetic nicotine via the use of heavy metal catalysts in the synthetic steps (Stephens et al., 2005). Consumption of lead can lead to a range of health issues, especially neurotoxic effects, developmental problems in children, and cardiovascular effects in adults. Even low levels of lead exposure over time can have detrimental health effects. Arsenic is a known carcinogen, and elevated levels of arsenic consumption can lead to skin, lung, bladder, and other cancers. Additionally, it can cause other health issues such as cardiovascular disease and skin lesions which are more dangerous than nicotine itself (Pappas et al., 2014). Monitoring lead and arsenic levels is part of quality control measures to ensure the consistency and safety of nicotine-containing products.

**FIGURE 6:**
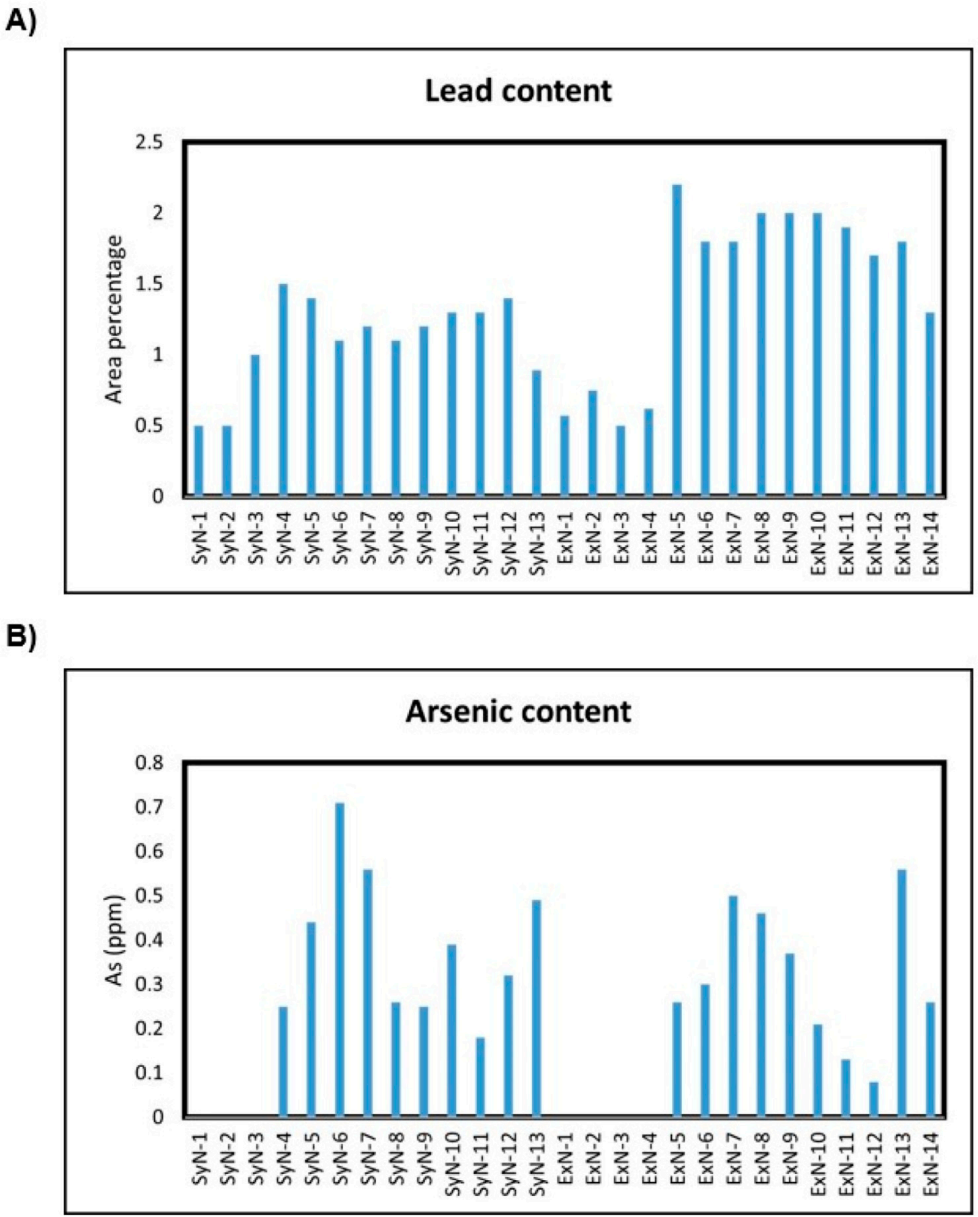
**(A)** Pb and **(B)** As amounts (ppm) in synthetic and extracted nicotine lots.

See the Supplementary Section S2 for the calculation process, lead and arsenic linearity curves, and absorbance results for each nicotine sample. The tabulated results for Pb and As in parts per million (ppm) for all the nicotine lots are shown in Figure 6.

## Discussion and conclusion

Synthetic nicotine (SyN) has gained the interest of related product vendors over the past decade mainly due to a regulatory loophole that came into effect last year, in 2023. It has been claimed by manufacturers and vendors that SyN is purer than tobacco-extracted nicotine (ExN) due to the absence of plant residues and is less harmful. However, none of the smoking product vendors list the purity and related impurities in SyN products. Herein, a thorough evaluation of purity and impurities present in 13 Porton SyN lots in comparison to 14 ExN lots is presented for the awareness of smoking product consumers.

Both SyN and ExN lots exist in nicotine-free base form, above ∼99% assay results. The enantiomeric purity of the SyN was identical to ExN and is above 99.9% of (S)-Nicotine isomer. Cotinine, Myosamine, Nornicotine, and Nicotine N-oxide, were not present or present in low amounts compared to ExN lots making SyN nicotine lots purer. Anatabine, Anabasine, and β-Nicotyrine impurities were absent in both SyN and ExN lots. The metals, lead and arsenic were found to be equal in both SyN and ExN.

Nicotine-related nitrosamine content was also tested for both SyN and ExN lots and was not detected in any lots. It was expected that ExN to contain high percentages of nitrosamine impurities as these are naturally formed in Tobacco plants/leaves during growth. Nitrosamine content in ExN depends on the maturity of Tobacco leaves at the time of harvest where pre-mature leaves do not contain nitrosamine impurities. The formation of TSNAs is influenced by various factors, including the type of tobacco, curing methods, and the conditions under which tobacco is stored. The levels of TSNAs can vary among different tobacco products and brands. Therefore, the possibility of having nitrosamines in ExN cannot be fully eliminated.

In conclusion, the Nicotine from in-house SyN lots is identical to ExN in terms of assay and enantiomeric purity. The overall impurity content in SyN was low compared to ExN with few exceptions. The key advantage of SyN is that the Tobacco leaves harvest does not depend on weather conditions and saves the land for other important vegetation cultivation. Furthermore, extracted nicotine products are often overloaded with flavor to mitigate any smell or rustic flavor incorporated during harvesting (hay-like odor, bitter, and astringent taste). Synthetic nicotine does not require such flavoring due to the controlled nature of synthesis in a laboratory.

Additionally, it’s important to note that the health risks of tobacco use extend beyond the impurities/TSNAs quantified in this work, and tobacco smoke contains numerous other harmful substances that contribute to various diseases, including respiratory and cardiovascular conditions. As a result, the best approach to minimize health risks associated with tobacco is to avoid Tobacco either extraction or synthesis approach.

One of the main limitations of this study is the availability of various SyN and ExN types due to regulatory concerns. A statistical analysis could have been carried out if more lots were available. Future work includes improving the efficiency of the methods such as developing a single High-Resolution-Mass-Spectrometry (HRMS) method to identify and quantify both alkaloid impurities and nicotine nitrosamine rather than using two methods: HPLC and LC-MS.

## Data Availability

The original contributions presented in the study are included in the article/Supplementary Material, further inquiries can be directed to the corresponding authors.

## References

[B1] Avagyan R. Spasova M. Lindholm J. (2021). Determination of nicotine-related impurities in nicotine pouches and tobacco-containing products by liquid chromatography–tandem mass spectrometry. Separations 8 (6), 77. 10.3390/separations8060077

[B2] Cheetham A. G. Plunkett S. Campbell P. Hilldrup J. Coffa B. G. Gilliland S. (2022). Analysis and differentiation of tobaccoderived and synthetic nicotine products: addressing an urgent regulatory issue. PLoS One 17 (4), e0267049. 10.1371/journal.pone.0267049 35421170 PMC9009602

[B3] Duell A. K. Pankow J. F. Peyton D. H. (2018). Free-base nicotine determination in electronic cigarette liquids by 1H NMR spectroscopy. Chem. Res. Toxicol. 31 (6), 431–434. 10.1021/acs.chemrestox.8b00097 29775302 PMC6008736

[B4] El-Hellani A. El-Hage R. Baalbaki R. Salman R. Talih S. Shihadeh A. (2015). Free-base and protonated nicotine in electronic cigarette liquids and aerosols. Chem. Res. Toxicol. 28 (8), 1532–1537. 10.1021/acs.chemrestox.5b00107 26158618 PMC4920054

[B5] Gholap V. V. Heyder R. S. Kosmider L. Halquist M. S. (2020). An analytical perspective on determination of free base nicotine in E-liquids. J. Anal. Methods Chem. 2020, 1–12. 10.1155/2020/6178570 PMC7085884 32257508

[B6] Henningfield J. E. London E. D. Pogun S. (2009). Handbook of experimental pharmacology, Nicotine psychopharmacology. Heidelberg: Springer. Vol. 192. 19301485

[B7] Jordt S. E. (2023). Synthetic nicotine has arrived. Tob. Control. NLM (Medline) 32, e113–e117. 10.1136/tobaccocontrol-2021-056626 PMC8898991 34493630

[B8] Konstantinou E. Fotopoulou F. Drosos A. Dimakopoulou N. Zagoriti Z. Niarchos A. (2018). Tobacco-specific nitrosamines: a literature review. Food Chem. Toxicol. 118, 198–203. 10.1016/j.fct.2018.05.008 29751076

[B9] Kowitt S. D. Seidenberg A. B. Gottfredson O’Shea N. C. Ritchie C. Galper E. F. Sutfin E. L. (2023). Synthetic nicotine descriptors: awareness and impact on perceptions of e-cigarettes among US youth. Tob. Control. 10.1136/tc-2023-057928 PMC10640660 37173133

[B10] Lin W. (2023). Method for Synthesizing (S)-Nicotine. European Patent: EP 4119671A1. Publication Number: WO 2021/180019, France.

[B11] Lisko J. G. Tran H. Stanfill S. B. Blount B. C. Watson C. H. (2015). Chemical composition and evaluation of nicotine, tobacco alkaloids, PH, and selected flavors in E-cigarette cartridges and refill solutions. Nicotine Tob. Res. 17 (10), 1270–1278. 10.1093/ntr/ntu279 25636907 PMC4573955

[B12] Lisuma J. B. Mbega E. R. Ndakidemi P. A. (2019). Influence of nicotine released in soils to the growth of subsequent maize crop, soil bacteria and fungi. Int. J. Agric. Biol., 1–12. 10.17957/IJAB/15.1026

[B13] Ma S. Qiu Z. Chen J. Shang C. (2023). Synthetic nicotine E-liquids sold in US online vape shops. Prev. Med. Rep. 1, 102222. 10.1016/j.pmedr.2023.102222 PMC10172710 37181243

[B14] Pankow J. F. Barsanti K. C. Peyton D. H. (2003). Fraction of free-base nicotine in fresh smoke particulate matter from the eclipse “cigarette” by 1H NMR spectroscopy. Chem. Res. Toxicol. 16 (1), 23–27. 10.1021/tx020050c 12693027

[B15] Pappas R. S. Fresquez M. R. Martone N. Watson C. H. (2014). Toxic metal concentrations in mainstream smoke from cigarettes available in the USA. J. Anal. Toxicol. 38 (4), 204–211. 10.1093/jat/bku013 24535337 PMC4540051

[B16] Robinson J. H. Pritchard W. S. (1992). The role of nicotine in tobacco use. Psychopharmacology (Berl) 108, 397, 407. 10.1007/bf02247412 1410152

[B17] Stephens W. E. Calder A. Newton J. (2005). Source and health implications of high toxic metal concentrations in illicit tobacco products. Environ. Sci. Technol. 39 (2), 479–488. 10.1021/es049038s 15707047

[B18] Talhout R. Schulz T. Florek E. van Benthem J. Wester P. Opperhuizen A. (2011). Hazardous compounds in tobacco smoke. Int. J. Environ. Res. Public Health 8 (2), 613–628. 10.3390/ijerph8020613 21556207 PMC3084482

[B19] Talih S. Salman R. El-Hage R. Karaoghlanian N. El-Hellani A. Saliba N. (2020). Effect of free-base and protonated nicotine on nicotine yield from electronic cigarettes with varying power and liquid vehicle. Sci. Rep. 10 (1), 16263. 10.1038/s41598-020-73385-6 33004992 PMC7530983

[B20] Yalcin E. de la Monte S. (2016). Tobacco nitrosamines as culprits in disease: mechanisms reviewed. J. Physiology Biochem. 72, 107–120. 10.1007/s13105-016-0465-9 PMC4868960 26767836

[B21] Zafeiridou M. Hopkinson N. S. Voulvoulis N. (2018). Cigarette smoking: an assessment of tobacco’s global environmental footprint across its entire supply chain. Environ. Sci. Technol. 52 (15), 8087–8094. 10.1021/acs.est.8b01533 29968460

