# Supplementary material for: Comparison of genotoxic impurities in extracted nicotine vs. synthetic nicotine
Source: Front Chem. 2024 Oct 14;12:1483868. doi: 10.3389/fchem.2024.1483868 (PMC11514277; doi:10.3389/fchem.2024.1483868)
Supplement: Supplementary file 1 [file DataSheet1.docx]

*Supporting information to accompany:*

**Comparison of Genotoxic Impurities in Extracted Nicotine vs. Synthetic Nicotine**

**Ayesha Nisathar^1^, Hui Chen^1^, Xiaoli Lei^2^, Zeyu Zeng^2^, Jia Chen^2^**

1. **JSTAR Research Inc./Porton USA: Analytical Research Development and Quality control: 6 Cedarbrook Drive, Cranbury, New Jersey 08512, USA**
2. **Porton Pharma Solutions: Room 701, 7^th^ Floor, Building 11, No.88, Keyuan South Road, Gaoxin District, Chengdu City, Sichuan 610093, China and No.7, Yuntu Road, Beibei District, Chongqing, 400799, China**

Table of Contents

1. Nitrosamine impurity identification method and results
2. Metal analysis results
3. References
4. **Nitrosamine impurity identification and quantification method and results**

**1.1 Nitrosamine testing method**

LCMS-MRM (multiple reaction monitoring) from Nicotine regulation protocol, China was used for qualitative and quantitative analysis of nitrosamines. The tested N-nitrosamines are N'-Nitrosonornicotine (NNN), 4-(Methylnitrosamino)-1-(3-pyridyl)-1-butanone (NNK), N-Nitrosoanatabine (NAT), and N-Nitrosoanabasine (NAB). Deuterated N-Nitroso Nicotine (NNN-d4), Deuterated 4-sulfur nitrosamine) -1-(3-pyridinyl) -1-butone (NNK-d4), Deuterated Nitrosonornitine (NAT-d4), and deuterated (R, S)-N-Nitrostobasine (NAB-d4) was used as internal standards for NNN, NNK, NAT, and NAB respectively. Liquid chromatography (LC) parameters) and mass spectrometry (MS) parameters used are listed in Table S1 and S2, respectively.

**Table S 1.** LC parameters for nitrosamine testing.

| Instrument | Agilent 6470 with 1290 Triple Quadrupole LC/MS) | | |
| --- | --- | --- | --- |
| Column | Infinity Lab Poroshell 120 EC-C18 100×4.6mm, 4μm | | |
| Mobile phase A | 10 mmol/L Ammonia acetate solution | | |
| Mobile phase B | 0.1% acetic acid in MeCN | | |
| Flow rate | 0.4 mL/min | | |
| Diluent | 0.1 mol/L ammonia acetate | | |
| Inj. volume | 5 μL | | |
| Sample tray temperature | Room Temp | | |
| Column temperature | 30 °C | | |
| Ion source | ESI | | |
| Fragmentor | 100 V | | |
| Nebulizer pressure | 45 psi | | |
| Gradient | Time (min) | A (%) | B (%) |
|  | 0 | 90 | 10 |
|  | 2 | 60 | 40 |
|  | 4 | 40 | 60 |
|  | 6 | 10 | 90 |
|  | 9 | 10 | 90 |
|  | 9.5 | 90 | 10 |
|  | 15 | 90 | 10 |

**Table S 2.** MS parameters for nitrosamine testing.

| Compound | Ion pair (collision energy/eV) | |
| --- | --- | --- |
|  | Quantitative | Qualitative |
| NNN | 178.1>148.2 (15) | 178.1>120.1 (15) |
| NNN-d4 | 182.1>152.2 (15) | —— |
| NNK | 208.1>122.1 (16) | 208.1>106.1 (16) |
| NNK-d4 | 212.1>126.1 (16) | —— |
| NIGHT | 190.1>160.1 (15) | 190.1>106.1 (15) |
| NAT-d4 | 194.1>164.1 (15) | —— |
| NAB | 192.1>162.2 (17) | 192.1>133.1 (17) |
| NAB-d4 | 196.1>166.2 (17) | —— |

Preparation of testing of standard and sample solutions are described below.

- Diluent: 0.1 M ammonia acetate

7.70 g of ammonia acetate (77.98 g/mol, 0.1 mol) was weighed and transferred to a 1000 mL volumetric flask (VF) and diluted up to mark with Milli-Q water and mixed well to dissolve.

- Preparation of internal standard solution

Internal standard stock solution: 10.0 mg of each NNN-d4, NNK-d4, NAT-d4, NAB-d4 was accurately weighed and transferred to separate 10 mL amber VF, and dissolved with acetonitrile (MeCN), mixed well and diluted up to the mark to get 1 mg/mL each.

Mixed internal standard solution 1: 1.0 mL of each internal standard stock solution NNN-d4, NNK-d4, NAT-d4, NAB-d4 was transferred to a 100 mL amber VF. 50 mL of MeCN was added and dissolved well. Each solution was diluted up to the mark with MeCN to get 10 μg/mL of each internal standard.

Mixed internal standard solution 2: 10.0 mL of mixed internal standard solution was transferred 100 mL amber VF with 50 mL of MeCN, bring to mark with MeCN and mix well to get 1 μg/mL of each internal standard.

- Preparation of linearity curve standard solutions

Standard solutions:

Standard Stock Solution: 5.0 mg of NNN, NNK, NAT, NAB was accurately weighed and transfered to 5 mL of separate amber VF bring each of VF with MeCN to volumetric mark, swirl and mix well to get 1 mg/mL each.

Mixed standard solution 1: 1.0 mL of NNN, NNK, NAT each standard stock solution and 0.5 mL of NAB standard stock solution was transferred to an amber 100 mL VF with 50 mL of MeCN, bring to mark with MeCN and mix well to get 10 μg/mL of NNN, NNK, NAT standard and 5 μg/mL of NAB standard.

Mixed standard solution 2: 10.0 mL of mixed standard solution was transferred to 100 mL amber VF with 50 mL of MeCN, bring to mark with MeCN and mix well to get 1.0 μg/mL of NNN, NNK, NAT standard and 0.5 μg/mL of NAB Standard.

Nitrosamine linearity solutions LS-1, LS-2, LS-3, LS-4 and LS-5 were prepared by mixing ‘mixed internal standard solution 2’ (internal std-2) and ‘mixed standard solution 2’ (mixed std-2) according to the Table S3.

**Table S 3.** Nitrosamine linearity solution preparation volumes.

| Linearity solution ID | V _internal std-2_  (mL) | V _mixed std-2_  (mL) | Final volume  (mL) | C _NNN, NNK, NAT each_  (ng/mL) | C _NAB_  (ng/mL) |
| --- | --- | --- | --- | --- | --- |
| LS-1 | 0.1 | 0.1 | 100 | 1.0 | 0.5 |
| LS-2 | 0.1 | 0.5 | 100 | 5.0 | 2.5 |
| LS-3 | 0.1 | 1.0 | 100 | 10.0 | 5.0 |
| LS-4 | 0.1 | 2.0 | 100 | 20.0 | 10.0 |
| LS-5 | 0.1 | 5.0 | 100 | 50.0 | 25.0 |

- Sample preparation

1.0g of each nicotine sample was accurately weighed and placed into a 15 mL centrifuge tube with a stopper, and 100µL of the mixed internal standard solution 2 was added, adjusted the volume to 10 mL with 0.1 mol/L ammonium acetate solution (diluent). The centrifuge tube was sealed with a cap and placed it in a vortex shaker and vortexed at 2000 r/minute for 30 minutes. The extraction solution was injected according to LCMS-MRM conditions. Each nitrosamine impurity was quantified based on the standard curve.

**1.2. Nitrosamine test results in each nicotine lot**

Nitrosamine retention times are listed in Table S5, and the standard curve was prepared based on the response of each nitrosamine according to Table S4. Linearity equations for each nitrosamine are shown in Table S6.

**Table S 4.** Response of each nitrosamine at each concentration.

| C _NNN, NNK, NAT each_  (ng/mL) | Response _NNN_ | Response _NNK_ | Response _NAT_ | C _NAB_  (ng/mL) | Response _NAB_ |
| --- | --- | --- | --- | --- | --- |
| 1.0 | 1639 | 2029 | 1464 | 0.5 | 1272 |
| 5.0 | 8778 | 10985 | 8447 | 2.5 | 7441 |
| 10.0 | 19044 | 24039 | 18716 | 5.0 | 16300 |
| 20.0 | 40395 | 53210 | 41114 | 10.0 | 35473 |
| 50.0 | 106476 | 141689 | 109482 | 25.0 | 94900 |

**Table S 5.** LC retention times of each nitrosamine and each deuterated nitrosamine.

| Compound | Retention time (minutes) | Compound | Retention time (minutes) |
| --- | --- | --- | --- |
| NNN | 7.230 | NNN-d4 | 7.214 |
| NNK | 7.443 | NNK-d4 | 7.431 |
| NAT | 7.773 | NAT-d4 | 7.754 |
| NAB | 7.856 | NAB-d4 | 7.835 |

**Table S 6.** Linearity equations for each nitrosamine.

| Compound name | Linearity equation | Concentration Range (ng/mL) | r^2^ |
| --- | --- | --- | --- |
| NNN | y=1.190508x-0.134828 | 1-50 | 1.0000 |
| NNK | y=1.021843x-0.24113 | 1-50 | 0.9999 |
| NIGHT | y=1.07253x+0.648702 | 1-20 | 0.9987 |
| NAB | y=0.882235x-0.002647 | 0.5-25 | 1.0000 |

A sample chromatogram from LS-1 linearity solution is shown in Figure S1.

**Figure S 1.** Nitrosamine standard chromatogram for LS-1

A sample nitrosamine chromatogram for synthetic nicotine lot, SyN-1 and extracted nicotine, ExN-1 is shown in Figure S2 and S3, respectively.


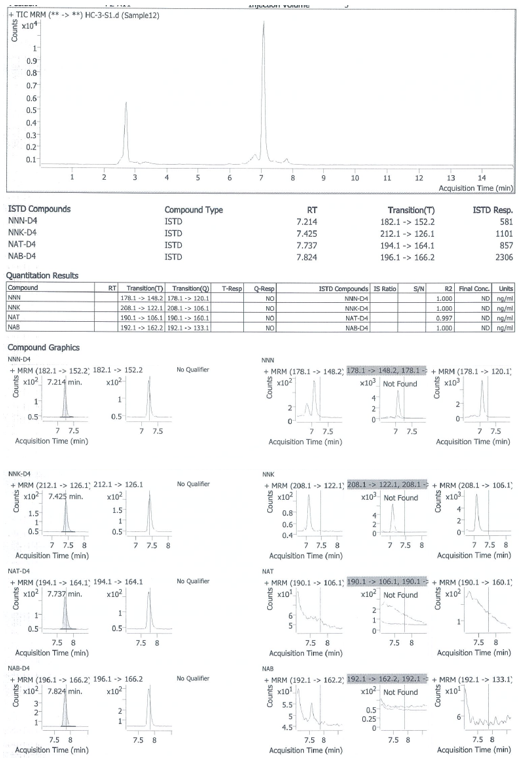


**Figure S 2.** Example Nitrosamine chromatogram and respective TICs for a synthetic nicotine sample, SyN-1


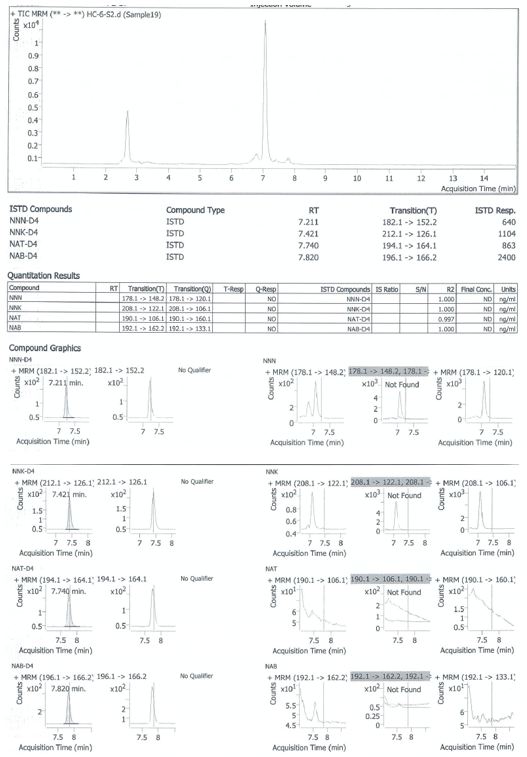


**Figure S 3.** Example Nitrosamine chromatogram and respective TICs of an extracted nicotine sample, ExN-1.

Nitrosamine testing for each synthetic and extracted nicotine was done in duplicate and nitrosamines were not detected in any sample.

1. **Metal analysis**
   1. **Pb analysis**

Atomic absorption spectrophotometer SP-3803AA， Shanghai Spectral Instrument Co., Ltd instrument was used for Pb analysis in nicotine samples. Instrument parameters are listed in the Table S7 below.

**Table S 7.** Atomic absorption spectrometer parameters for Pb analysis.

| The 2013-201 | Graphite furnace |
| --- | --- |
| Background correction | Deuterium lamp background correction |
| Wavelength | 283.30nm |
| bandwidth | 0.7nm |
| High voltage | 240.2 volts |
| Work light current | 4.0mA |
| Atomization heating method | Optical |
| Drying and ashing time | 144 second |
| Atomization time | 4 second |
| Atomization temperature | 1900℃ |
| Calculation method | Peak height |
| Computing time | 4.0 second |
| Repetitions | 1 |

Pb analysis for all the nicotine samples were done in two separate batches. SyN-1 to SyN-3 and ExN-1 to ExN-4 was tested one as one batch using a one Pb standard curve. Pb standard concentrations and absorbance are for the first standard curve is shown in Table S8 and the linearity plot in Figure S4. Pb content calculation results for the first batch are shown in Table S9. SyN-4 to SyN-13 and ExN-5 to ExN-14 nicotine samples were tested separately for Pb content analysis. The standard curve results and the standard curve for the second batch is shown in Table S10 and Figure S5, respectively. The Pb analysis calculation and Pb content for nicotine samples in the second batch is tabulated in Table S11.

**Table S 8.** Pb standard curve-1 test results.

| Concentration (µg/L) | Absorbance |
| --- | --- |
| 0 | 0.002 |
| 2 | 0.0311 |
| 5 | 0.0605 |
| 10 | 0.1153 |
| 15 | 0.1781 |

**Figure S 4.** Pb standard curve 1.

Pb content in ppm was calculated using the equation below,

Pb (ppm) = *10$\frac{C*v*n}{m}$^-3^

C: pb concentration in the sample (µg/L), V: final volume (25 mL), n: Dilution factor, m: Sample weight (g).

**Table S 9.** Nicotine samples Pb testing results for batch 1.

| Sample ID | Sample weight  (g) | Final volume  (mL) | Dilution factor | Absorbance | Pb concentration  (μg/L) | Pb content (ppm) |
| --- | --- | --- | --- | --- | --- | --- |
| SyN-1 | 0.1100 | 25 | 1 | 0.027 | 2.009 | 0.46<0.5 |
| SyN-2 | 0.1100 |  |  | 0.0278 | 2.078 | 0.47<0.5 |
| SyN-3 | 0.1174 |  |  | 0.0599 | 4.875 | 1.04 |
| ExN-1 | 0.1217 |  |  | 0.036 | 2.793 | 0.58 |
| ExN-2 | 0.1138 |  |  | 0.043 | 3.403 | 0.75 |
| ExN-3 | 0.1129 |  |  | 0.0217 | 1.547 | 0.34<0.5 |
| ExN-4 | 0.1144 |  |  | 0.0378 | 2.95 | 0.64 |

**Table S 10.** Pb standard curve-2 test results.

| Pb concentration  (µg/L) | Absorbance |
| --- | --- |
| 0 | 0.0031 |
| 2 | 0.0152 |
| 5 | 0.037 |
| 10 | 0.0701 |
| 15 | 0.1018 |
| 20 | 0.1307 |

**Figure S 5.** Pb standard curve 2.

**Table S 11.** Nicotine samples Pb testing results for batch 2.

| Sample ID | Sample weight  (g) | Final volume  (mL) | Dilution factor | Absorbance | Pb concentration  (μg/L) | Pb content (ppm) |
| --- | --- | --- | --- | --- | --- | --- |
| SyN-4 | 0.2285 | 25 | 1 | 0.0908 | 13.498 | 1.5 |
| SyN-5 | 0.2157 |  |  | 0.0795 | 11.746 | 1.4 |
| SyN-6 | 0.2117 |  |  | 0.0649 | 9.481 | 1.1 |
| SyN-7 | 0.2061 |  |  | 0.0658 | 9.621 | 1.2 |
| SyN-8 | 0.2103 |  |  | 0.0635 | 9.264 | 1.1 |
| SyN-9 | 0.2051 |  |  | 0.0652 | 9.528 | 1.2 |
| SyN-10 | 0.2078 |  |  | 0.0734 | 10.799 | 1.3 |
| SyN-11 | 0.2203 |  |  | 0.0762 | 11.234 | 1.3 |
| SyN-12 | 0.2121 |  |  | 0.0819 | 12.118 | 1.4 |
| SyN-13 | 0.2220 |  |  | 0.0541 | 7.806 | 0.87 |
| ExN-5 | 0.2227 |  |  | 0.1300 | 19.579 | 2.2 |
| ExN-6 | 0.2128 |  |  | 0.1027 | 15.344 | 1.8 |
| ExN-7 | 0.2325 |  |  | 0.1098 | 16.445 | 1.8 |
| ExN-8 | 0.2134 |  |  | 0.1132 | 16.973 | 2.0 |
| ExN-9 | 0.2011 |  |  | 0.1083 | 16.213 | 2.0 |
| ExN-10 | 0.1954 |  |  | 0.1058 | 15.825 | 2.0 |
| ExN-11 | 0.2086 |  |  | 0.1063 | 15.903 | 1.9 |
| ExN-12 | 0.1964 |  |  | 0.0905 | 13.452 | 1.7 |
| ExN-13 | 0.1993 |  |  | 0.0963 | 14.351 | 1.8 |
| ExN-14 | 0.2020 |  |  | 0.0739 | 10.877 | 1.3 |

- 1. **As analysis**

Atomic fluorescence spectrophotometer AFS-933 Beijing Jitian Instrument Co., Ltd was used for Arsenic (As) analysis in nicotine samples. Instrument method parameters are listed below in Table S12.

**Table S 12.** Atomic fluorescence spectrometer parameters for As analysis.

| Element | Channel A: None.  Channel B: As |
| --- | --- |
| Negative high voltage of photomultiplier tube | 270 V |
| Atomizer height | 8 mm |
| lamp current | Channel A:0 mA.  Channel B:60 mA |
| carrier gas flow rate | 400 mL/min |
| Shield gas flow rate | 800 mL/min |

Analysis of As for all the synthetic and extracted nicotine was done in two separate batches similar to Pb analysis. All the standard curve results, standard curves along with As calculations are shown below (As standard curve 1: see Table S 13 and Figure S 6, As standard curve 2: see Table S 15 and Figure S 7, As content in nicotine lots in Table S 14 and Table S 16).

**Table S 13.** As standard curve-1 test results.

| Concentration (µg/L) | Fluorescence |
| --- | --- |
| 0 | 0 |
| 1.000 | 51.84 |
| 2.000 | 102.57 |
| 5.000 | 237.41 |
| 10.000 | 513.74 |

**Figure S 6.** As standard curve 1.

**Table S 14.** Nicotine samples As testing results batch 1.

| Sample ID | Sample weight  (g) | Final volume  (mL) | Dilution factor | Fluorescence | As concentration  (μg/L) | As content (ppm) |
| --- | --- | --- | --- | --- | --- | --- |
| SyN-1 | 0.1100 | 25 | 1 | 23.58 | 0.508 | 0.12 |
| SyN-2 | 0.1158 |  |  | 18.29 | 0.404 | 0.09 |
| SyN-3 | 0.1174 |  |  | 24.64 | 0.528 | 0.11 |
| ExN-1 | 0.1217 |  |  | 37.88 | 0.788 | 0.16 |
| ExN-2 | 0.1138 |  |  | 46.84 | 0.964 | 0.21 |
| ExN-3 | 0.1129 |  |  | 44.49 | 0.918 | 0.20 |
| ExN-4 | 0.1225 |  |  | 27.96 | 0.593 | 0.12 |

**Table S 15.** As standard curve-1 test results.

| As concentration  (µg/L) | Fluorescence |
| --- | --- |
| 0 | 0 |
| 2.000 | 304.52 |
| 5.000 | 715.43 |
| 10.000 | 1367.89 |
| 15.000 | 2025.40 |
| 20.000 | 2591.61 |

**Figure S 7.** As standard curve 2.

**Table S 16.** Nicotine samples As testing results batch 2.

| Sample ID | Sample weight  (g) | Final volume  (mL) | Dilution factor | Fluorescence | As concentration  (μg/L) | As content (ppm) |
| --- | --- | --- | --- | --- | --- | --- |
| SyN-4 | 0.2285 | 25 | 1 | 304.09 | 2.014 | 0.25 |
| SyN-5 | 0.2157 |  |  | 538.24 | 3.819 | 0.44 |
| SyN-6 | 0.2117 |  |  | 821.06 | 5.998 | 0.71 |
| SyN-7 | 0.2061 |  |  | 643.35 | 4.628 | 0.56 |
| SyN-8 | 0.2103 |  |  | 330.15 | 2.215 | 0.26 |
| SyN-9 | 0.2051 |  |  | 312.02 | 2.076 | 0.25 |
| SyN-10 | 0.2078 |  |  | 459.12 | 3.209 | 0.39 |
| SyN-11 | 0.2203 |  |  | 245.24 | 1.561 | 0.18 |
| SyN-12 | 0.2121 |  |  | 396.36 | 2.725 | 0.32 |
| SyN-13 | 0.2220 |  |  | 620.05 | 4.449 | 0.49 |
| ExN-5 | 0.2227 |  |  | 341.26 | 2.301 | 0.26 |
| ExN-6 | 0.2128 |  |  | 379.90 | 2.599 | 0.30 |
| ExN-7 | 0.2325 |  |  | 640.28 | 4.605 | 0.50 |
| ExN-8 | 0.2134 |  |  | 557.04 | 3.963 | 0.46 |
| ExN-9 | 0.2011 |  |  | 425.18 | 2.947 | 0.37 |
| ExN-10 | 0.1954 |  |  | 251.33 | 1.608 | 0.21 |
| ExN-11 | 0.2086 |  |  | 179.05 | 1.051 | 0.13 |
| ExN-12 | 0.1964 |  |  | 126.44 | 0.646 | 0.082 |
| ExN-13 | 0.1993 |  |  | 625.57 | 4.491 | 0.56 |
| ExN-14 | 0.2020 |  |  | 315.41 | 2.102 | 0.26 |

1. References
2. Shen Y.; Zhang, N.; Prinyawiwatkul, W.; Xu, Z. A rapid LC-MS/MS method for simultaneous determination of nicotine and its key derivatives including hydroxylation isomers: Int J. Mass Spectrom. 2021, 468, 116642

https://doi.org/10.1016/j.ijms.2021.116642
